# Case series: Four fatal rabbit hemorrhagic disease virus infections in urban pet rabbits

**DOI:** 10.3389/fvets.2023.1144227

**Published:** 2023-03-22

**Authors:** Filipe Fontes Pinto, Joana Abrantes, Paula Gomes Ferreira, Mário Nóbrega, Ricardo Marcos

**Affiliations:** ^1^HIPRA, Malveira, Portugal; ^2^UMIB—Unit for Multidisciplinary Research in Biomedicine, ICBAS—School of Medicine and Biomedical Sciences, University of Porto, Porto, Portugal; ^3^CIBIO—Centro de Investigação em Biodiversidade e Recursos Genéticos, InBIO Laboratório Associado, Universidade do Porto, Vairão, Portugal; ^4^BIOPOLIS Program in Genomics, Biodiversity and Land Planning, CIBIO, Vairão, Portugal; ^5^Departamento de Biologia, Faculdade de Ciências, Universidade do Porto, Porto, Portugal; ^6^ITR—Laboratory for Integrative and Translational Research in Population Health, University of Porto, Porto, Portugal; ^7^ICBAS—School of Medicine and Biomedical Sciences, University of Porto, Porto, Portugal; ^8^Exoticvets, Loures, Portugal

**Keywords:** rabbit hemorrhagic disease, RHD, rabbit hemorrhagic disease virus, RHDV, case series

## Abstract

Four pet rabbits (*Oryctolagus cuniculus cuniculus*) diagnosed with a fatal infection by rabbit hemorrhagic disease virus (RHDV GI.2) were identified in the same week and further investigated. All animals lived in an urban environment (Lisbon, Portugal), were between 8 months and 2 years old and none had been vaccinated against RHDV2 (GI.2). Three animals arrived at the clinic and died shortly afterward and it was only possible to collect material for RT-qPCR (RHDV) test. These rabbits tested positive for RHDV2, with high viral loads. In the fourth case, additional clinical and post-mortem gross and histological evaluations were performed. This 8 month old intact female indoor pet rabbit was presented with apathy, tachypnea and tachycardia. Radiographic projections revealed no clinical revealed no clinical abnormalities. Serum biochemistry revealed a significant increase in AST and ALT with a small hypoglycemia. Abdominal ultrasound revealed an acute hepatitis. Despite hospitalization support, after 30 h of admission, the rabbit lost consciousness and developed anorexia and pyrexia in the last minutes before death. Post-mortem analysis and molecular testing by RT-qPCR, confirmed the diagnosis of RHDV2 (GI.2) infection also with high viral load. In conclusion, this paper reports a case series that demonstrates the severe infectious ability and the high mortality associated with RHDV even in rabbits from urban environments. Furthermore, it highlights the importance of always considering rabbit hemorrhagic disease (RHD) as a differential diagnosis in pet rabbits with non-specific clinical signs, and should warn veterinarians that pet rabbits living indoors can also be infected with a fatal outcome.

## Introduction

The emergence of infectious disease outbreaks can impact our life and lead to negative outcomes in the economy and environment. The European rabbit is a keystone species of the Mediterranean ecosystems as it is the main prey for several predators; in addition, it is a relevant meat source, and has gained increased interest as a pet animal. It is affected by two viral diseases, myxomatosis and rabbit hemorrhagic disease (RHD), which are good examples of how viral outbreaks can impact different rabbit-related sectors.

RHD is a highly infectious disease caused by a non-enveloped RNA virus belonging to the *Caliciviridae* family, the rabbit hemorrhagic disease virus (RHDV) (1–3). Due to its high mortality and morbidity rates, the virus has had a huge impact since its emergence in the 1980s (4). According to a proposed nomenclature, the RHDV pathogenic strains can be grouped into two genotypes: GI.1 (known as classic RHDV) and GI.2 (also known as new variant or RHDV2/b) (5).

The classic RHDV (GI.1) was first reported in 1984 (4) and induces an acute or hyperacute disease in adult rabbits, characterized by a fulminant liver failure and hemorrhagic diathesis. In contrast, GI.2, which emerged in 2010 (6), also affects rabbits younger than 6–8 weeks old and has been associated with different clinical courses and with a far broader host range (6–8). Since the first outbreaks, several GI.2 recombinant strains have been described (8–10), with a rapidly spread across the world, and affecting both wild and farm rabbits (11–16).

In Portuguese wild rabbit populations, GI.2 outbreaks seem to be related with the breeding season (November-May), when more new young rabbits susceptible to infection are available (15). Additionally, GI.2 infections have also been reported in farm rabbits (16). Despite this, the prevalence of GI.2 infection in pet rabbits is unknown with only scarce cases reported to date, and a lack of in-depth epidemiological investigation of the circulating strains.

The natural infection of RHDV is still poorly understood (17–20). Blood-feeding insects have been shown to be effective mechanical vectors, and all possible transmission routes are considered, including oral, nasal, conjunctival, and parenteral (1). RHDV can be transmitted directly by infected rabbits' secretions and excretions, or indirectly through fomites—contaminated food, bedding, water, clothing, cages, and equipment, or by vector-borne transmission (1). Liver damage plays a key role in the pathogenesis of RHD (21).

## Case description

In the first week of December 2021, four pet rabbits from different owners were presented to an exotic veterinary center in Lisbon, Portugal. The first three cases (rabbits 1–3; Table 1) were presented with apathy and tachypnea, dying moments after admission. Therefore, it was only possible to perform their necropsy and collect material for molecular tests. A fourth case allowed a more in-depth clinical investigation.

**Table 1:** General information of the pet rabbits.

**Rabbit n°**	**Gender**	**Age (months)**	**Accommodation**	**Vaccination status**	**Other information**	**RHDV2 RT-qPCR result**
# 1	Male	11	Urban, exclusively indoor	Unknown	None	Positive Ct 23.2
# 2	Female	24	Urban, exclusively indoor	Unknown	It had a litter and all the offspring died 2 days later	Positive Ct 24.6
# 3	Male	17	Urban, indoor with garden access	Classical RHD and myxomatosis	None	Positive Ct 24.5
# 4	Female	8	Urban, exclusively indoor	Classical RHD and myxomatosis	Ultrasound compatible with acute hepatitis and increased AST and ALT levels	Positive Ct 22.3

This fourth case was an 8-month-old intact female pet rabbit (*Oryctolagus cuniculus cuniculus*) that was referred due to apathy and tachypnea. This rabbit had been vaccinated against classic RHD and myxomatosis and dewormed with selamectin and fenbendazole 4 months before admission (August 2021). Its diet included *ad libitum* hay, 50 grams of morning feed, and a variety of vegetables and mid-afternoon fruit. In addition, the rabbit was occasionally fed with fresh grass from natural city parks. Apart from a its previous visit to another veterinary center, the animal was always kept indoors.

On physical examination, the animal was in good body condition. In addition to apathy and tachypnea, the rabbit also had tachycardia, but its rectal temperature was normal (39°C; reference range: 38.5–40°C) (22). Ventrodorsal and lateral radiographic projections were obtained from the rabbit skull, thorax, and abdomen, but no abnormalities were observed. Two hours after hospitalization, an abdominal ultrasound revealed a brightness and greater exposure of the portal wall veins, with a generally reduced liver echogenicity, compatible with acute hepatitis. A complete blood count was performed with an automated analyzer (Mindray BC-2800 Vet Auto Hematology Analyzer, Mindray, China) which turned out to be normal (22). However, a slight increase in young heterophils and activated lymphocytes were detected on the microscopic evaluation of blood smear. Serum biochemistry was performed with Abaxis VetScan VS2 (profile plus) and revealed a significant increase in AST (1741 U/L; Ref 14-113 U/L) (22) and ALT (621 U/L; Ref 14-80 U/L) (22) with a small hypoglycemia (59 mg/dL; Ref 75-150 mg/dL) (22).

Despite being hospitalized to reverse its clinical condition, the rabbit developed anorexia and pyrexia, which intensified in the last minutes before death, 30 h after admission. In the seconds preceding death, the animal lost consciousness and made a loud vocalization. The clinical history and the antemortem findings suggested that the rabbit might had an infectious and/or inflammatory process that led to non-specific clinical signs and a sudden death. Therefore, numerous differential diagnoses were considered, including sepsis, poisoning, RHD, heatstroke, enteric disease, epizootic rabbit enteropathy, Tyzzer's disease, hepatic coccidiosis, neoplasia, respiratory disease, aspiration pneumonia, trauma, liver or lung lobe torsion, and gastrointestinal stasis, dilatation or obstruction.

**Figure 1:**
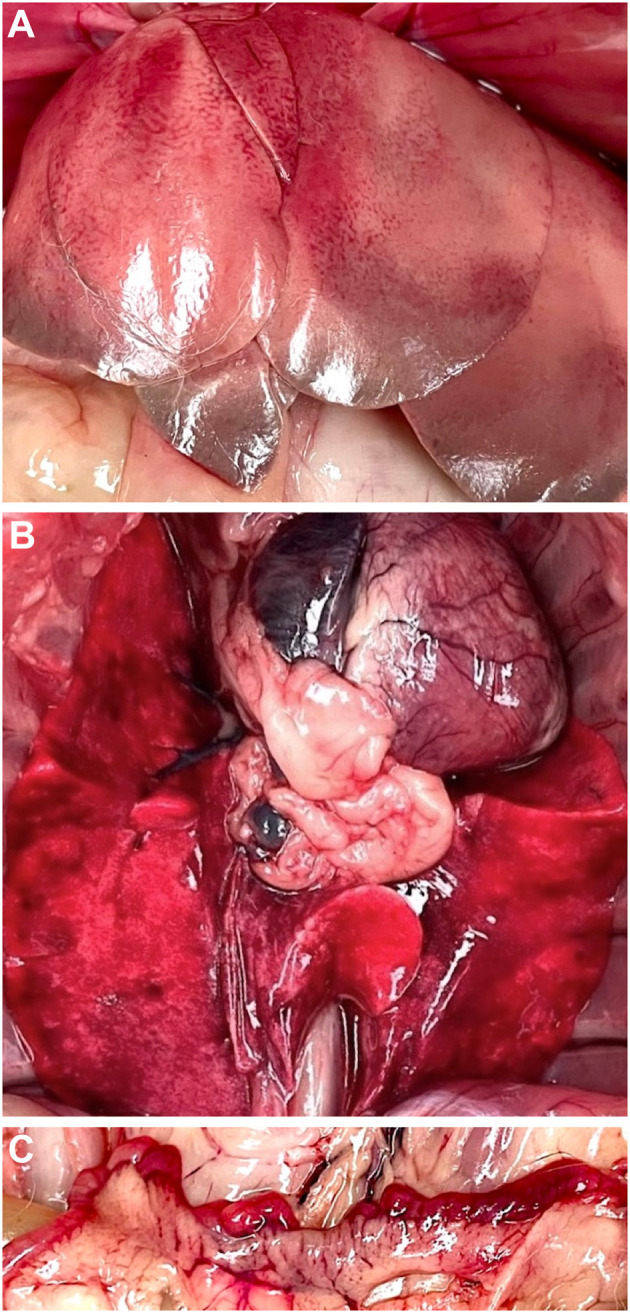
Necropsy findings in rabbit #4 (*Oryctolagus cuniculus cuniculus*) case. Liver with an enlarged, pale, and distinct lobular pattern with light gray discoloration in the edges of the lobes (likely a postmortem change) **(A)**. Lungs **(B)** and uterus **(C)** had multifocal to coalescing hemorrhages.

**Figure 2:**
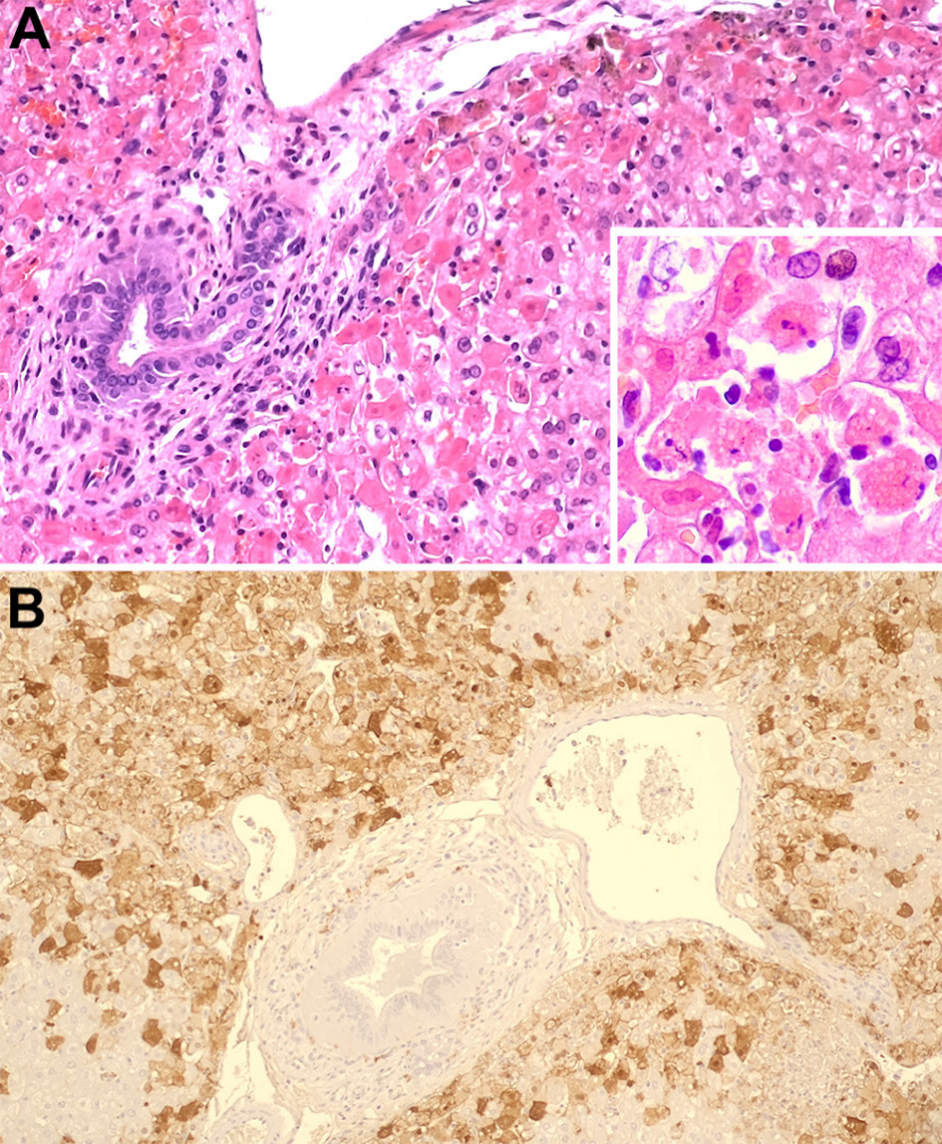
Liver with a severe generalized hepatocellular necrosis with hemorrhage, most evident in the periportal areas, H&E stain **(A)**. Liver, detail of necrotic cells, H&E [**(A)** inset]. Liver. Marked hepatocytes' immunolabeling with monoclonal antibody (Mab) against rabbit hemorrhagic disease virus capsid proteins **(B)**.

At necropsy, the liver had an enlarged, pale, and distinct lobular pattern with regional areas of light gray discoloration in the edges of all lobes (likely a postmortem change) (Figure 1A). Additionally, the lungs (Figure 1B) and uterus (Figure 1C) had multifocal to coalescing hemorrhages. The liver, kidneys, lungs and uterus were sampled, fixed in 10% formalin, and routinely processed for histological examination. Hepatocellular necrosis with hemorrhage was detected in all sections examined (Figure 2A), generally with a random distribution, although more evident in the periportal areas. Mononucleated inflammatory cells were observed near portal triads. The necrotic areas were sometimes associated with variable congestion and sinusoidal fibrin thrombi. Furthermore, the lungs and kidneys had hemorrhages and occasional fibrin thrombi in small vessels.

Immunohistochemistry was performed for RHDV antigen detection in the liver. For this, liver sections (3 μm thick) mounted on adhesive microscope slides were immunostained using the modified avidin–biotin–peroxidase complex method (23). Endogenous peroxidase was blocked by treatment with 3% hydrogen peroxide in methanol for 20 min. After washing 2 x 5 min in PBS with 0.05% Tween, the sections were incubated for 20 min in a moist chamber with normal rabbit serum (ref. X092, Dako, Glostrup, Denmark) diluted 1:5 in BSA 10%, to block the non-specific binding. The sections were then incubated overnight at 4°C with the monoclonal antibody (MAb) 1G5 diluted 1:10 in BSA 5%. MAb 1G5 was produced as described by Almanza et al. (24) and recognizes RHDV and RHDV2 capsid proteins as described by Bárcena et al. (25). Liver tissue was then incubated for 30 min with a biotin-labeled rabbit anti-mouse secondary antibody (ref. EO35301-2, Dako, Glostrup, Denmark) diluted 1:200 in BSA 5% followed by incubation in an avidin-biotin-peroxidase complex (Vector Laboratories, Peterborough, UK) diluted 1:100 in 5% BSA for additional 30 min. The slides were incubated with DAB substrate (3,3′-diaminobenzidine, Dako) during 5 min for color development. Sections were counterstained with Mayer's hematoxylin (ref. HX390929, Merck, Darmstadt, Germany) and dehydrated slides were mounted in Entellan. Figure 2B depicts the hepatocytes' positive staining with the immunohistochemical technique, confirming the presence of RHDV. Along with the clinical findings, the histopathologic and immunohistochemistry results were compatible with an infection by RHDV.

Liver samples of the four rabbits were sent to HIPRA Diagnos Laboratory, Amer (Girona), Spain, where they were analyzed by real-time RT-qPCR to determine the presence of RHDV and quantify viral loads. Briefly, the RNeasy Mini Kit (Qiagen, Germany) was used to extract and purify the RNA from the samples, according to the manufacturer's instructions. The RT-qPCR was performed with the primers described in Duarte et al. (26) using 35 ng of purified RNA. Amplification was carried out in 25 μL volume reactions using the QuantiTect Probe RT-PCR Kit (Qiagen, Germany), according to the manufacturer's recommendations, in a 7500 FAST real time system (Applied Biosystem). Primers (RHDV2-F 5′ TGGAACTTGGCITGAGTGTTGA 3′ and RHDV2-R 5′ ACAAGCGTGCTTGTGGACGG 3′) and probe (RHDV2 probe 5′ FAM-TGTCAGAACTTGTTGACATCCGCCC-TAMRA 3′) were used at a final concentration of 0.4 μM and 0.2 μM, respectively. Cycling conditions included one cycle at 50°C for 30′, one cycle at 95°C for 15′ and 40 cycles at 94°C for 15″ and 60°C for 30″. The sample Ct values were used to estimate template quantity and the calculations were performed using the 7500 Software version 2.0.6. The results obtained indicated that all rabbits tested GI.2 (RHDV2/b) positive (Table 1) and were associated with a high viral load. Therefore, a diagnosis of infection by RHDV GI.2 (RHDV2/b) was obtained for the four suspected rabbits.

## Discussion

The final diagnosis of RHD was confirmed by RT-qPCR for all rabbits and by additional histopathological and immunohistochemical evaluation for the fourth rabbit. In adult rabbits (older than 8 weeks-old), the classical RHDV (GI.1) infection often induces acute or hyperacute forms of the disease, characterized by a fulminant liver failure and hemorrhagic diathesis, and, more rarely, subacute or chronic forms (1, 27). RHDV2 (GI.2) also affects rabbits <6–8 weeks old and presents hyperacute, acute or chronic forms (6, 19, 27). Typical signs of RHD include mucosal congestion, neurological signs, cyanosis, dyspnea, foamy hemorrhagic epistaxis, and ocular hemorrhage (1). However, in the hyperacute form, the animals may die suddenly without previously showing any sign of disease (1). In the acute form, the clinical signs are frequently unspecific, as recently described in pet rabbits (28) and as observed herein. Likewise, in subacute/chronic forms, the animals show unspecific signs of disease, such as anorexia and apathy, but also jaundice, which is the most typical clinical sign. Since 2010, strains isolated from RHDV-positive animals belong to the GI.2 serotype. Chronic and subclinical courses are more common in GI.2 than in GI.1 (6, 19) making the diagnosis challenging. Although a reliable definitive diagnosis of RHD can only be made post-mortem, this disease should always be considered as a differential diagnosis in pet rabbits with non-specific clinical signs.

As shown with these four clinical cases, pet rabbits kept indoors are incorrectly considered to have a lower risk of RHDV infection. Indeed, RHDV represents a serious threat due to its high transmissibility and high stability in the environment (1). Transmission by insects or other fomites may have been the source of infection in these animals; in the fourth rabbit, the fed fresh grass collected from natural parks was an additional possible route of infection. While in this case the animal was vaccinated against RHDV (GI.1), it was not vaccinated against the current RHDV2 (GI.2) circulating strains, which caused the fulminant hepatitis. In Portugal, GI.2 circulates since 2012 and GI.1 was last detected in 2011. Yet, many rabbits are still referred to exotic veterinarians with inadequate vaccination protocols. It should be also noted that even rabbits properly vaccinated against GI.2 can still develop a fatal clinical disease (29). The high substitution rate of this virus (30), as observed for other RNA viruses, increases the chances of the fixation of mutations that allow immune escape. Therefore, veterinarians must be aware of immunization failures, either by animal immunity breaks or vaccine mismanagement, or changes in RHDV virulence. In addition, when an infected rabbit is admitted to a veterinary center, it poses a serious health threat to other animals due to the virus' highly contagious nature and environmental resistance. Therefore, when a positive case is confirmed, or even suspected, it is important to implement proper biosecurity measures in the handling and disposal of potentially RHDV-infected animals, as well their samples and/or excretions (feces and urine).

In Portugal, RHDV GI.2 outbreaks seem to be related to the breeding season (November-May) in wild rabbit populations; the four cases were diagnosed within this period, in the first week of December. Additional investigations are thus required for better understanding RHDV epidemiology and its relation with pet animals and their environment. In our opinion, all pet rabbits should be vaccinated against myxomatosis and RHD—GI.1 and GI.2, and RHDV vaccine boosters should be performed taking into account potential high risk periods.

In conclusion, this paper confirms the severe infectious nature and high mortality associated with RHDV, and emphasizes that outbreaks can occur even in urban environments and among pet rabbits. Furthermore, it highlights the need to always consider RHD as a differential diagnosis in pet rabbits with non-specific clinical signs, and should constitute a warning to all veterinarians that pet rabbits living indoors can also be infected with a fatal outcome.

## Data availability statement

The original contributions presented in the study are included in the article/supplementary material, further inquiries can be directed to the corresponding author.

## Ethics statement

Ethical review and approval was not required for the animal study. Written informed consent was obtained from the owners for the participation of their animals in this study.

## Author contributions

FP collected the data and drafted the manuscript. MN handled the cases in the clinic. JA, PF, and RM contributed to the cases interpretation and manuscript editing. All authors contributed to the final version of the manuscript.
